# Different Effect of Proteasome Inhibition on Vesicular Stomatitis Virus and Poliovirus Replication

**DOI:** 10.1371/journal.pone.0001887

**Published:** 2008-04-02

**Authors:** Nickolay Neznanov, Eugenia M. Dragunsky, Konstantin M. Chumakov, Lubov Neznanova, Ronald C. Wek, Andrei V. Gudkov, Amiya K. Banerjee

**Affiliations:** 1 Department of Molecular Genetics, Lerner Research Institute, Cleveland Clinic Foundation, Cleveland, Ohio, United States of America; 2 Center for Biologics Evaluation and Research, Food and Drug Administration, Rockville, Maryland, United States of America; 3 Department of Biochemistry and Molecular Biology, Indiana University School of Medicine, Indianapolis, Indiana, United States of America; 4 Department of Cell Stress Biology, Roswell Park Cancer Institute, Buffalo, New York, United States of America; 5 Cleveland Biolabs, Inc., Buffalo, New York, United States of America; Institut Pasteur, France

## Abstract

Proteasome activity is an important part of viral replication. In this study, we examined the effect of proteasome inhibitors on the replication of vesicular stomatitis virus (VSV) and poliovirus. We found that the proteasome inhibitors significantly suppressed VSV protein synthesis, virus accumulation, and protected infected cells from toxic effect of VSV replication. In contrast, poliovirus replication was delayed, but not diminished in the presence of the proteasome inhibitors MG132 and Bortezomib. We also found that inhibition of proteasomes stimulated stress-related processes, such as accumulation of chaperone hsp70, phosphorylation of eIF2α, and overall inhibition of translation. VSV replication was sensitive to this stress with significant decline in replication process. Poliovirus growth was less sensitive with only delay in replication. Inhibition of proteasome activity suppressed cellular and VSV protein synthesis, but did not reduce poliovirus protein synthesis. Protein kinase GCN2 supported the ability of proteasome inhibitors to attenuate general translation and to suppress VSV replication. We propose that different mechanisms of translational initiation by VSV and poliovirus determine their sensitivity to stress induced by the inhibition of proteasomes. To our knowledge, this is the first study that connects the effect of stress induced by proteasome inhibition with the efficiency of viral infection.

## Introduction

Proteasomes are cellular structures responsible for rapid, efficient and strictly regulated process of protein degradation [1]. The substrates of degradation are first subjected to poly-ubiquitination and then digested by the proteasomes [2]. The ubiquitin/proteasome pathway is the major route for regulated protein degradation in eukaryotic cells [1]. Besides special targets for ubiquitination and proteasome-specific degradation, such as p53, IκBα, STAT [3]–[6], proteasomes are responsible for the degradation of unfolded or improperly folded proteins [7], [8]. In combination with chaperones, this activity of proteasomes is important for maintaining cellular protein homeostasis [9], [10].

Proteasome specific degradation is an important part of replication of several viruses [11], [12]. Some viruses developed mechanisms to target cellular proteins, such as p53 and STAT, for proteasome-specific degradation [13], [14]. The stability of some viral proteins, including poliovirus and HAV protein 3C, also depends on the proteasome activity [15]. In the present work, we studied the role of proteasomes in the replication of vesicular stomatitis virus (VSV) and poliovirus.

VSV belongs to the Rhabdoviridae [16]. Rhabdoviruses have negative strand RNA genomes, and their replication begins with synthesis of positive strand mRNAs by viral RNA polymerase [17]. Similar to cellular mRNAs, VSV mRNAs are capped and polyadenylated [18], [19]. Replicating virus efficiently competes with cellular processes for the substrates of RNA and protein synthesis and for the translational machinery [20]–[23]. As a result, virus proteins represent a sizeable share of newly synthesized proteins in the infected cells.

Poliovirus belongs to the Enterovirus genus of the Picornaviridae [24]. Picornaviruses have positive-strand RNA genomes that can be translated immediately after infection [25]. Unlike VSV and most cellular mRNAs, the picornavirus translation initiation occurs at an internal ribosome entry site (IRES) [26]. Poliovirus replication triggers cleavage of eIF4G, which is needed for cap-dependent initiation, leading to suppression of cellular protein synthesis and rapid accumulation of viral proteins and RNA [27], [28].

Ubiquitination has broad effects on viral infections. For example, the ability of ubiquitination to regulate endocytosis and endosomal membrane transport [29] may contribute to the maturation and budding of retroviruses [30], [31] and paramyxoviruses [32], including Rhabdoviruses, such as VSV and rabies virus [32], [33]. The role of free ubiquitin and proteasomes in the late stage of VSV replication, including budding, was previously postulated [33].

In contrast to the effects of proteasome/ubiquitin activities on VSV maturation and budding [33], we found a new effect of proteasome inhibition on VSV protein synthesis and virus accumulation. This effect is evident during early steps of VSV replication. Proteasome inhibition had a detrimental effect on VSV and cellular protein synthesis in virus-infected cells, and protected the cells from the toxic effect of VSV infection. Negative effect of proteasome inhibitors on VSV replication and cellular protein synthesis was not detected in GCN2−/− cells. In contrast, poliovirus replication was less sensitive to the effect of proteasomes inhibitors. Inhibition of proteasome activity delayed all processes during poliovirus replication, but did not abrogate them, and did not change virus accumulation or its toxic effect. We suggest that stress induced by accumulation of aberrant translation products following proteasome inhibition leads to the suppression of cap-dependent translation, explaining why proteasome inhibitors block replication of VSV but not poliovirus replication.

## Results

### Inhibition of proteasome activity suppresses VSV replication and protects the cells from infection

**Figure 1:**
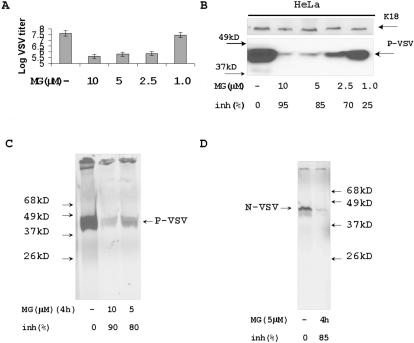
Proteasome inhibitor MG132 suppresses VSV replication and protein synthesis. (A) Titration of VSV in MG132 treated cells. HeLa cells were infected with VSV (MOI = 1) for one hour with additional washing and incubated overnight. Medium from control VSV infected cells and VSV infected cells treated with 10 µM, 5 µM, 2.5 µM, and 1 µM of MG132 were used for plaque assay to detect virus replication. Results represent average data of two experiments. (B) Western blotting with anti-P-protein antibodies. HeLa cells were infected with VSV (MOI = 5) for 1 hour. After changing the medium, MG132 was added in the indicated concentrations and the cells were incubated for additional 4 h. Total protein extracts (5 µg) were analyzed with anti-P-protein Abs. Keratin 18 (K18) was a protein loading control. Intensity of each band was estimated with ImageJ software to calculate percentage of viral protein synthesis inhibition. (C) Immunoprecipitation of S^35^ labeled P-protein. HeLa cells were infected with VSV (MOI = 5) for 4 h. Proteins were labeled with S^35^ methionine/cysteine for last 30 min of infection and VSV P-protein was precipitated with specific antibodies from cytoplasmic protein extracts and analyzed by electrophoresis and autoradiography. MG132 was added for 4 h in indicated concentrations. (D) Immunoprecipitation of S^35^ labeled N-protein. The protein extracts described in panel C were precipitated with antibodies specific to N-protein. Proteins were analyzed by electrophoresis and autoradiography.

To study the effect of proteasome inhibitor on VSV replication, HeLa cells were treated with various amounts of proteasome inhibitor MG132 (Calbiochem) and simultaneously infected with VSV overnight. Low concentrations of the inhibitor (1 to 10 µM) did not have a visible toxic effect on cells after overnight incubation. While infection with VSV at MOI = 1 led to complete destruction of untreated HeLa cells overnight, addition of MG132 in the indicated concentrations protected the cells from the toxic effect of VSV infection. Reduced virus yield confirmed that MG132 suppressed VSV replication in HeLa cells at concentrations starting from 2.5 µM (Fig. 1A). To analyze VSV protein synthesis, HeLa cells were infected with VSV at MOI = 5 for 4 h and treated with various amounts of MG132 at a time of infection. Reduced levels of VSV protein were also observed by immunoblotting (Fig. 1B).

Additional experiments were performed by using immunoprecipitation of S^35^ methionine/cysteine-labeled proteins from VSV infected cells. HeLa cells were infected with VSV at MOI = 5 for 4 h and treated with various amount of MG132 at a time of infection. The newly synthesized proteins were labeled by S^35^-methionine and cysteine, and cytoplasmic protein extracts were prepared according to Dingmans' protocol [34]. Next, VSV proteins P and N were precipitated from the cytoplasmic extracts using respective antibodies. The efficiency of virus protein synthesis was assessed by electrophoresis and autoradiography (Fig. 1 C, D). A significant concentration-dependent suppression of virus replication and viral protein synthesis by 5 and 10 µM of MG132 was observed in all experiments.

### The time course analysis of MG132 inhibitory activity

**Figure 2:**
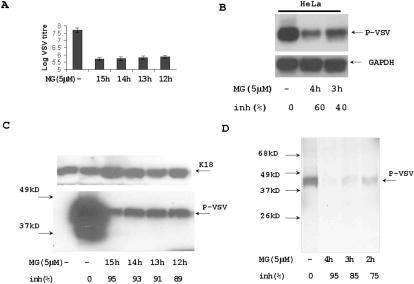
The effect of MG132 on VSV replication at different time of infection. (A) Titration of VSV virus from medium of overnight infected HeLa cells. HeLa cells were infected with VSV MOI = 1. The incubation of the cells with virus lasted one hour with additional washing. 5 µM of MG132 were added to cells at time of infection (15 h), 1 h (14 h), 2 h (13 h), and 3 h (12 h) after VSV infection. Results represent average data of two experiments. (B) VSV mRNA synthesis in MG132 treated cells. Northern blot analysis of 10 µg of total RNA from VSV (MOI = 5) infected for 4 h cells treated with MG132 at a time of infection (4 h), or 1 h after infection (3 h). Hybridization with P^32^ labeled P-protein cDNA probe. RNA loading was standardized by hybridization with GAPDH-gene probe. The hybridization signal of each band was estimated by ImageJ software to calculate percentage of RNA synthesis inhibition. (C) Immunoblotting with anti P-protein Abs. HeLa cells were infected with VSV (MOI = 1) and treated with 5 µM of MG132 as indicated in panel A. Total protein extracts (5 µg) from these cells were purified and tested by Western blotting with anti-P-protein Abs. Keratin 18 was a protein loading control. (D) Immunoprecipitation of S^35^-methionine labeled P-protein from VSV infected cells. HeLa cells were infected with VSV (MOI = 5) and treated with 5 µM of MG132 at time of infection (4 h), 1 h after infection (3 h), or 2 h after infection (2 h). After 4 h of infection the cells were incubated with S^35^-methionine/cysteine for 30 min. Cytoplasmic protein extracts were purified and VSV P-protein was precipitated with anti-P-protein Abs. The efficiency of P-protein synthesis was estimated by electrophoresis and autoradiography.

In our original experiments, we treated VSV-infected cells with MG132 at the start of infection. In the next experiment, HeLa cells were treated with 5 µM of MG132 at time of infection, one, two, and three hours after VSV infection to analyze the connection between the effect of proteasome inhibitor and virus internalization. The efficiency of virus replication after an overnight infection with VSV at MOI = 1 (Fig. 2A, C) or after 4 h at MOI = 5 (Fig. 2 B, D) was evaluated by plaque assay (Fig, 2A), by Northern (Fig. 2B), by Western blotting of 5 µg of total protein with anti-P-protein antibodies (Fig. 2 C), and by immunoprecipitation of S^35^-labeled P-protein (Fig. 2 D). Although the inhibiting effect of MG132 on VSV replication was strongest when the drug was added earlier, significant inhibition of VSV replication was still detectable when MG132 was added 3 h after VSV infection.

### Detrimental effect of proteasome inhibition on VSV replication does not depend on the inhibitor type

**Figure 3:**
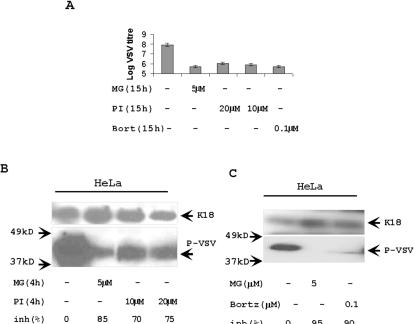
Different proteasome inhibitors affect VSV replication. (A) Proteasome inhibitor 1 and Bortezomib decreased VSV replication. Titration of VSV from the medium of overnight infected HeLa cells. VSV infection (MOI = 1) for one hour was substituted by the regular medium with indicated concentration of proteasome inhibitors. VSV was titrated by plaque assay after overnight growth. (B) Analysis of P-protein synthesis in the cells treated with proteasome inhibitor 1. HeLa cells were infected with VSV (MOI = 5) for 4 h and treated with proteasome inhibitor 1 (PI) or MG132 (MG) at a time of VSV infection. The total protein extracts (5 µg) from these cells were analyzed by Western blotting with anti-P-protein Abs. The concentrations of proteasome inhibitors varied from 5 to 20 µM. Keratin 18 (K18) was a protein loading control. (C) Bortezomib suppressed VSV replication. HeLa cells were infected with VSV, treated with Bortezomib (100 nM) and MG132 (5 µM), and analyzed as described in panel B. K18 was a protein loading control.

To confirm that inhibitory effect of MG132 is due to its suppression of proteasome activity, we tested different proteasome inhibitors. Proteasome inhibitor 1 is a modified tri-peptide with a structure different from MG132. It proteasome-inhibiting activity requires higher concentrations than MG132. In these experiments MG132 served as a positive control. Both proteasome inhibitors affected VSV replication in HeLa cells in a similar manner (Fig. 3 panels A and B). Bortezomib (PS341) is a specific inhibitor of proteasomes, approved by FDA as anti-cancer drug [35]. Its structure and mechanism of action is different from MG132 and proteasome inhibitor 1. It is more active than MG132 and was used at concentration as low as 100 nM. Bortezomib, like other proteasome inhibitors, suppressed VSV replication (Fig. 3 panels A and C).

### Proteasome inhibitors delay the synthesis of poliovirus proteins, RNA, and the accumulation of live virus

**Figure 4:**
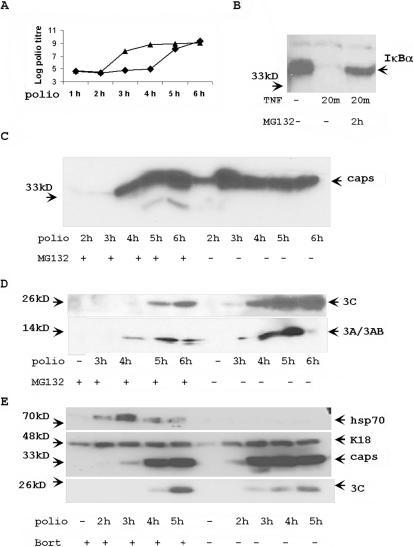
Proteasome inhibitors delay the replication of poliovirus. (A) HeLa cells (triangles) and HeLa cells pre-treated for 2 h with 5 µM proteasome inhibitor MG132 (squares) were infected with poliovirus strain Mahoney (MOI = 5) for 1 h. After replacement of medium, the accumulation of virus in medium was estimated by titration. (B) MG132 inhibits TNF-specific degradation of IκBα. Control HeLa cells and HeLa cells pretreated with 5 µM MG132 for 2 h were incubated with 1 ng/ml of human TNF for 20 min. 10 µg of total protein extracts were analyzed with anti-IκBα Abs. (C) HeLa cells and HeLa cells pre-treated with MG132 were infected with poliovirus (MOI = 5) for 1 h. After medium replacement, protein extracts were collected at different times of infection. The accumulation of poliovirus capsid proteins was tested in Western blotting experiments from 10 µg of protein extracts. (D) The protein extracts described in section B were tested with anti-proteins 3C and 3A Abs. The accumulation of poliovirus proteins 3C, 3A and 3AB were detected in 10 µg of protein extracts. (E) Bortezomib treatment attenuated poliovirus replication. HeLa cells were pretreated with Bortezomib for 2 h, then infected and analyzed as described in panels C and D. K18 was a loading control. Hsp70 is a control of Bortezomib activity.

**Figure 5:**
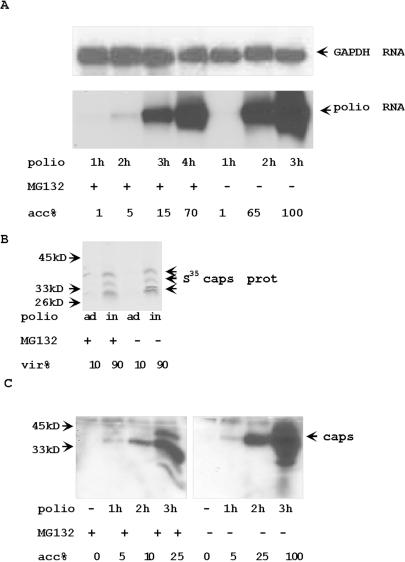
(A) The accumulation of poliovirus RNA was delayed but not abolished in MG132 treated poliovirus-infected cells. Northern blot hybridization of 5 µg of total RNA from poliovirus infected cells with poliovirus protein 3C hybridization probe. Hybridization with GAPDH gene was a RNA loading control. (B) The inhibition of proteasome activity does not affect the entrance of poliovirus into the cells. MG treated and control HeLa cells were pre-incubated with S^35^-labeled poliovirus (MOI = 100) for 1 h at 4°C. To estimate adsorption background, cells (ad) were washed with cold PBS. Virus internalization (in) was estimated by accumulation of S^35^-labeled poliovirus capsid proteins during additional 1 h incubation at 37°C. S^35^-labeled proteins were analyzed by electrophoresis and autoradiography. (C) Poliovirus capsid proteins accumulate slower in MG132 pretreated cells. The extracts from poliovirus-infected cells were analyzed with anti-poliovirus capsid Abs. Control or MG132 2 h pretreated cells were incubated with poliovirus (MOI = 5) for 1 h. Virus containing medium was washed out and cells were incubated for indicated time. 10 µg of protein from infected cells were analyzed by Western blotting with anti-poliovirus capsid Abs.

To understand the possible role of proteasomes in poliovirus replication, we studied kinetics of virus infection by titration infectious virus released into the medium of poliovirus-infected HeLa cells with and without two-hour MG132 pre-treatment. Although virus titers during late phases of viral infection (5–6 h) were similar in control cells and in cells pre-treated with MG132, virus accumulation was noticeably delayed between 3 and 4 h in cells, in which proteasome activity was suppressed with MG132 (Fig. 4A). The efficiency of proteasome inhibitor was confirmed by observing stabilization of IκBα in TNF-treated HeLa cells (Fig. 4B) [36]. As an independent confirmation of the changes in the kinetic of poliovirus accumulation in cells pre-treated with proteasome inhibitor, we found that the appearance and accumulation of poliovirus capsid proteins was similarly delayed in cells pre-treated with MG132 (Fig. 4C). In the same way, the appearance of viral non-structural proteins 3C and 3A/3AB was also delayed by one hour in MG132 treated poliovirus-infected cells (Fig. 4D). Bortezomib had similar effect on poliovirus replication (Fig. 4E). In agreement with the immunoblotting data, as assessed by Northern blot there was a delay in the accumulation of viral genomic RNA in MG132 pretreated cells (Fig. 5A).

### Inhibition of proteasome activity does not affect poliovirus cell entry

The delay of poliovirus replication in MG132 treated cells could be the result of less efficient entry of poliovirus into HeLa cells treated with MG132. To study the efficiency of the entry of poliovirus into HeLa cells, we tested for the presence of S^35^-labeled poliovirus capsid proteins in HeLa cells after incubation of HeLa cells with S^35^-labeled poliovirus. In this experiment, HeLa cells were incubated with poliovirus for one hour at 4°C, the virus-containing medium was removed, and cells were incubated for additional hour at 37°C. Proteins from cells were analyzed for S^35^-labeled virus capsid proteins by electrophoresis and autoradiography. A similar amount of capsid proteins from infecting virus could be detected during the first hour of infection regardless of whether cells were treated with proteasome inhibitor (Fig. 5B). Later during infection, capsid proteins accumulated sooner in control cells than in MG132-treated cells (Fig. 5C)

### Poliovirus-specific proteolytic cleavage of p65-RelA and eIF4G proteins is delayed by a proteasome inhibitor

**Figure 6:**
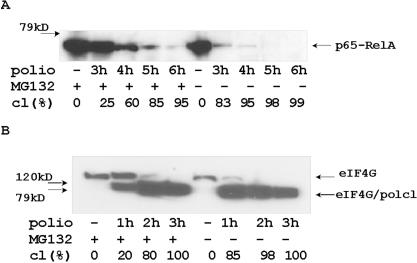
Proteolytic cleavage of p65-RelA and eIF4G occurred later during poliovirus infection of the cells with inhibited proteasome activity. HeLa cells and MG 132 2 h pretreated HeLa cells were infected with poliovirus (MOI = 5) for 1 h. After change of medium, total protein extracts were collected every hour and tested with anti-p65-RelA C-terminus specific Abs (A) or with anti eIF4G N-terminus specific Abs (B). 10 µg of protein were tested in Western blotting experiments.

Recently, we described the ability of poliovirus to cleave the p65-RelA subunit of NFκB transcription factor near its C-terminus [37]. This cleavage is protease 3C-specific and takes place between 2 and 3 h of infection in HeLa cells. The suppression of proteasome activity delayed the p65-RelA protease 3C-specific cleavage by about 60 min (Fig. 6A). These data indicate that the proteasome inhibitor MG132 did not stop the process of protease 3C-specific cleavage of p65-RelA in poliovirus-infected cells. Also, addition of MG132 to uninfected cells did not result in measurable increase of p65-RelA level. This suggests that slowing of p65-RelA degradation in poliovirus-infected cells treated with the proteasome inhibitor could be explained by the delay in synthesis of viral protease 3C that mediates this degradation.

The ability of the poliovirus protease to cleave the eIF4G translational initiation factor is an important step in the viral replicative cycle [38], [39]. This mechanism allows poliovirus to compete with cellular mRNAs for translation machinery and to inhibit many cellular defense responses that require synthesis of new antiviral proteins. The process of eIF4G degradation is one of the earliest events in poliovirus infection, occurring during first 90 min of infection in HeLa cells and depending on poliovirus protease 2A activity. Treatment of poliovirus-infected HeLa cells with a proteasome inhibitor delayed cleavage of p220 eIF4G protein (Fig. 6B). Thus, inhibition of proteasomes delayed, but did not abrogate virus specific cleavage of cellular proteins.

### Proteasome inhibition delays synthesis of poliovirus proteins

**Figure 7:**
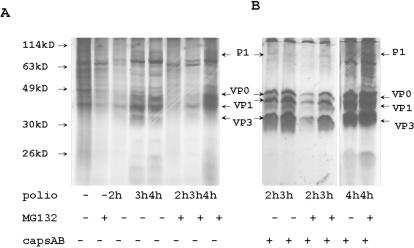
Poliovirus protein synthesis was delayed in MG132-pretreated cells. Control HeLa cells and HeLa cells pre-treated with MG132 for 2 h were infected with poliovirus (MOI = 5) for 2, 3 and 4 h. All cells were incubated in methionine/cysteine free medium supplemented with S^35^-methionine/cysteine for last 30 min before harvesting. To study general translation, 10 µg of cytoplasmic protein extracts were separated by electrophoresis and analyzed by autoradiography (A). To study poliovirus capsid protein accumulation, capsid proteins were precipitated by specific Abs from 100 µg of cytoplasmic protein extracts and analyzed by electrophoresis and autoradiography (B).

Accumulation of virus capsid protein and non-capsid proteins 3C, 3A, and 3AB was delayed in MG132 treated cells (Fig. 4 C, D). To study the process of poliovirus protein synthesis in more detail, HeLa cells were infected with poliovirus for 2, 3, and 4 h, and S**^35^** methionine/cysteine was added for 30 min at the end of each period. Capsid proteins were detected by immunoprecipitation, electrophoresis and then autoradiography (Fig. 7A, B). Again, the accumulation of de novo synthesized capsid proteins was delayed but not eliminated in MG132 treated cells.

No additional accumulation of capsid protein precursor P1 was detected in MG132 treated cells, indicating that MG132 did not suppress the activities of poliovirus proteases.

### The effect of inhibition of proteasome activity on cellular translation

**Figure 8:**
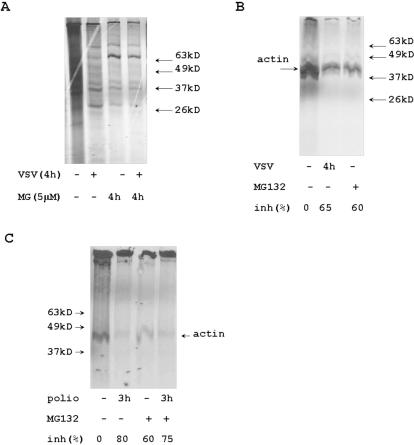
The effect of MG132 and virus infection on cellular protein synthesis. (A) Protein extracts were purified from control HeLa cells, cells infected with VSV for 4 h, cells treated with 5 µM of MG132 for 4 h, and cells infected with VSV and treated with MG132 for 4 h. All cells were incubated with S^35^ methionine/cysteine for last 30 min before the protein extracts purification. Cytoplasmic protein extracts were analyzed by electrophoresis and autoradiography. (B) Cytoplasmic protein extracts from control, VSV infected, and MG treated cells were precipitated with anti-actin Abs, and the complexes were purified on protein A agarose. S^35^ labeled actin was analyzed by electrophoresis and autoradiography. (C) Cytoplasmic S^35^-labeled protein extracts from MG-treated and poliovirus-infected cells were precipitated with anti-actin Abs and analyzed as described in panel B. All protein bands' intensity was detected by ImageJ software to calculate percentage of protein synthesis inhibition.

VSV and poliovirus infections suppress translation of cellular RNAs [40]–[43]. To analyze the translational activity in the cells treated with MG132 and infected with VSV or poliovirus, we labeled cellular proteins in vivo with S^35^ methionine/cysteine and immunoprecipitated the actin with specific antibodies (Fig. 8 A, B). Infection of HeLa cells with VSV and poliovirus decreased general cellular protein synthesis (Fig. 8A, 7A). Treatment of HeLa cells with MG132 suppressed cellular protein synthesis (Fig. 8A, 7A). This effect was also confirmed by the analysis of actin synthesis in MG132 treated cells (Fig. 8 B, C). In contrast to cellular protein synthesis, the accumulation of poliovirus capsid proteins was only slightly delayed in HeLa cells pre-treated with MG132 (Fig. 7B).

### Proteasome inhibition stimulates accumulation of chaperone hsp70 and the phosphorylation of eIF2α

**Figure 9:**
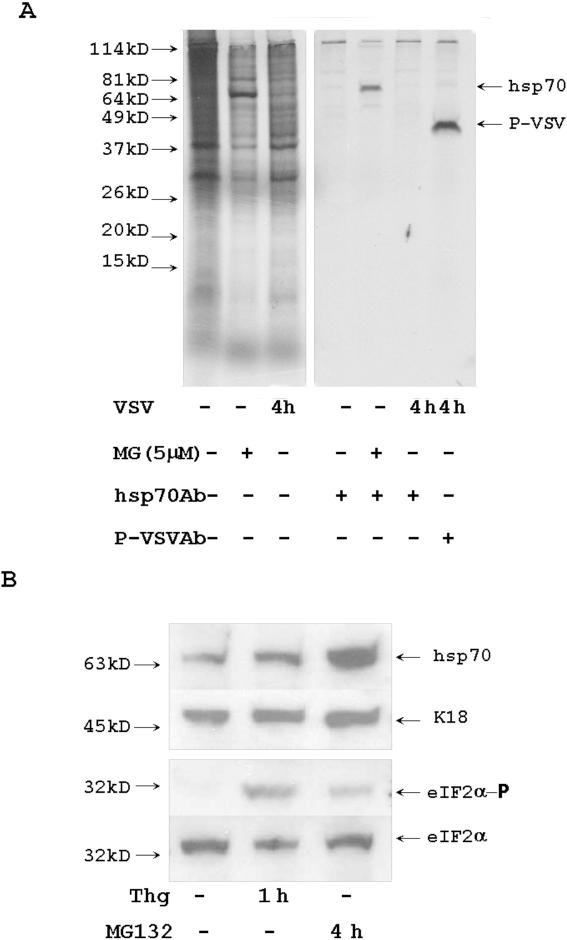
Treatment with MG132 activates stress. (A) Inhibition of proteasome activated hsp70 synthesis. Control HeLa cells, cells treated for 4 h with MG132, and 4 h VSV-infected cells were incubated for last 30 min with S^35^ methionine/cysteine. Cytoplasmic proteins were precipitated with anti-hsp70 and anti-P-VSV Abs. Precipitated proteins were analyzed by electrophoresis and autoradiography. (B) MG132 stimulated eIF2α phosphorylation. HeLa cells were treated with 1 µM of thapsigargin for 1 h and with 5 µM of MG132 for 4 h. 10 µg of protein extracts were analyzed with Abs specific for eIF2α and eIF2α- phosphate (eIF2α-P). Hsp70 is a marker of MG132 activated stress. Keratin 18 (K18) is a loading control.

Inhibition of general translation is a common consequence of various stress stimuli [44], [45]. Phosphorylation of eIF2α and accumulation of chaperone molecules are additional markers of the stress response [46]. We analyzed the appearance of these markers in HeLa cells treated with MG132. S^35^ pulse-labeled cytoplasmic protein extracts were purified and precipitated with anti-hsp70 antibodies for examination by electrophoresis and autoradiography. The results of these experiments are presented in Fig. 9 A. A newly synthesized 70 kD protein corresponding to hsp70 accumulated in all cells treated with MG132. Phosphorylation of eIF2α in MG132 treated cells was detected with antibodies specific to the phosphorylated form of eIF2α. Phosphorylation of eIF2α was detected in MG132-treated cells and in cells treated with another stress-inducing agent thapsigargin (an inhibitor of a sarco-endoplasmic reticulum Ca^+2^ ATPase), but was not detected in control HeLa cells (Fig. 9B). Thus, inhibition of proteasomes stimulated the appearance of stress markers, such as inhibition of general translation, phosphorylation of eIF2α, and the accumulation of chaperone hsp70.

### GCN2 activity is important for detrimental effect of proteasome inhibitor on VSV replication

**Figure 10:**
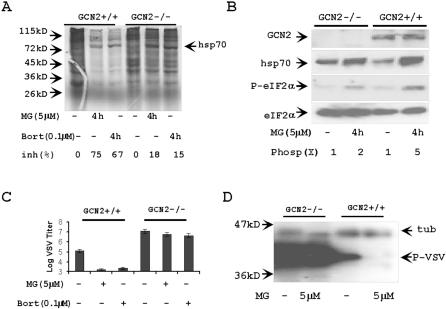
Inhibition of VSV replication in MG132 treated fibroblasts depends on GCN2. (A) Attenuation of translation in MG132- (MG), and Bortezomib (Bort) -treated cells is GCN2-dependent. Control wt GCN2+/+ MEF and GCN2−/− MEF, or cells treated with proteasome inhibitors for 4 h were incubated with S^35^-methionine/cysteine for 30 min. Protein synthesis was estimated by electrophoresis and autoradiography. (B) Western immunoblotting analysis of GCN2-dependent phosphorylation of eIF2α in response to MG132. 10 µg of protein extracts from control and MG132 treated cells were analyzed with indicated antibodies. Efficiency's fold of eIF2α phosphorylation (Phosp(x)) was estimated with ImageJ software. (C, D) Replication of VSV was not affected by proteasome inhibitors in GCN2−/− MEF. Proteasome inhibitors were added 1 h after infection with VSV (MOI = 1) and cells were incubated over night. Replication of VSV was estimated by titration in two experiments (C), or by Western immunoblotting with anti P-VSV protein Abs (D). Tubulin (tub) is a protein loading control.

GCN2 is a protein kinase responsible for eIF2α phosphorylation in response to amino acid starvation and some other stresses [44], [47]. Proteasome inhibitors' induced stress and attenuated translation is GCN2-dependent [44], [47]. To prove the role of stress induced by proteasome inhibition in suppression of VSV replication, we used wild type (wt) GCN2+/+ MEF and GCN2−/− MEF for VSV infection. Inhibition of proteasome activity attenuated general translation in wt GCN2+/+ MEF, but did not have effect on protein synthesis in GCN2−/− cells (Fig. 10A). MG132-specific phosphorylation of eIF2α factor was less efficient in GCN2−/− than in wt GCN2+/+ MEF cells (Fig. 10B). In agreement with these data, VSV replication was affected by inhibitors of proteasomes in wt GCN2+/+ MEF, but was not changed in GCN2−/− cells (Fig. 10 C, D). Surprisingly, GCN2 activity suppressed VSV replication in MEF without additional treatment with proteasome inhibitors (Fig. 10 C, D).

### Phosphorylation of eIF2α during VSV and poliovirus infection

**Figure 11:**
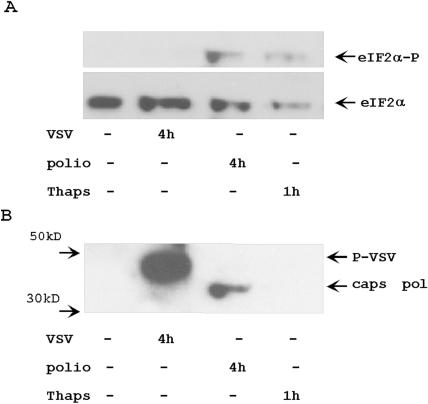
Different activation of eIF2α phosphorylation by VSV and poliovirus infections. HeLa cells were infected with VSV for 4 h, infected with poliovirus for 4 h, or treated with 1 µM of thapsigargin for 1 hour. Cytoplasmic protein extracts from these and control cells were analyzed with Abs against eIF2α and phosphorylated form of eIF2α (panel A). Same membrane was analyzed with Abs against VSV P- protein and poliovirus capsid proteins (panel B).

Virus infection is often connected with stress-related cellular processes, including induction of PKR- specific phosphorylation of eIF2α by double stranded viral RNAs [44], [48]. We analyzed the ability of poliovirus and VSV to activate eIF2α phosphorylation at 4 h after infection (Fig. 11). Poliovirus infection stimulated phosphorylation of translation initiation factor at 4 h after infection. In contrast, VSV infection did not induce phosphorylation of eIF2α at this time of infection. Our data coincide with earlier reports [49], [50]. According to these publications, eIF2α phosphorylation may be detected in poliovirus infected cells starting from 3 h post-infection, but in VSV infected cells eIF2α phosphorylation was detected only after 8 h of infection [49], [50]. Thus, there is a correlation between the ability of viral infection to stimulate eIF2α phosphorylation and its resistance to this phosphorylation stimulated by other stimuli, such as proteasome inhibitors.

## Discussion

Ubiquitination is important in the budding of retroviruses and Paramyxoviruses such as Sendai virus, VSV, and rabies virus [30], [32], [33]. Replication of coxsackieviruses was sensitive to inhibitor of proteasomes [51]. During late steps of Paramyxovirus replication, there is a decrease in accumulation of infectious virus in culture medium, but no change in the efficiency of virus protein synthesis with high concentrations of MG132 (up to 100 µM) [32], [33]. In this work, we describe novel effects of proteasome inhibitors on the replication of VSV and poliovirus, and demonstrate that these viruses differently respond to the inhibition. We present data indicating that the proteasome inhibition by 5 µM of MG132 ,10 µM of proteasome inhibitor 1, and 100 nM of Bortezomib at the early steps of VSV infection suppressed virus accumulation more than 100 fold and affected the synthesis of VSV proteins P and N. Proteasome inhibition decreased the efficiency of VSV replication when was administrated at the time of infection, as well as after it, indicating that the effect of MG132 on VSV replication does not depend on VSV entry into the cells. Treatment of VSV infected cells with MG132 slightly decreased the efficiency of VSV mRNA synthesis, which may be a consequence of the less efficient synthesis of VSV RNA polymerase L. This decline in the amount of RNA leads to further decrease in the amount of viral proteins.

In contrast to VSV, inhibition of proteasomes in HeLa cells delayed all processes during poliovirus replication by 60 to 90 min, but did not abolish the accumulation of poliovirus. Our data contrast with the effect of proteasome inhibitors on coxsackievirus replication in cardiomyocytes published by Luo H. [51]. The authors analyzed virus accumulation and synthesis of proteins and RNA at 7 h after infection, but did not analyze the kinetics of coxsackievirus replication. One explanation is that inhibitors of proteasomes may also delay coxsackievirus replication in cardiomyocytes, because 7 h of coxsackievirus replication may correspond to 3 h of poliovirus replication in HeLa cells. A longer observation period would be needed to see the effect. The alternative explanation may be the use of different cell lines for virus growth. Cardiomyocytes may respond differently to proteasome inhibition than do HeLa cells, resulting different effect on the efficiency of viral replication.

Proteasome-specific degradation of cellular proteins is an important mechanism for regulation of numerous cellular processes, including activation and inhibition of specifically regulated transcription and signal transduction, apoptosis, and the cell cycle [52]. In these processes, proteasomes are the essential components of the pathway that provides specific degradation of ubiquitinated substrates. Some viruses are able to target cellular proteins to proteasome-specific degradation [1], [53].

Another function of proteasomes is to maintain cellular protein homeostasis by degrading improperly folded, partially folded, or unfolded proteins [54]. Significant part of newly synthesized cellular proteins cannot fold correctly during synthesis. These unfolded proteins are called defective ribosome products [55]. Cells have two systems that deal with protein misfolding problem: the molecular chaperones and the ubiquitin-proteasome system [54]. The abundance of unfolded proteins increases under conditions when cells synthesize more proteins, for example during viral infection. The excess of newly synthesized unfolded proteins can lead to their aggregation with each other or other proteins. Translational attenuation is one of the responses to this stress [56], [57], and is mediated by phosphorylation of initiation factor eIF2α [47], [56], which is a common mechanism for regulation of protein synthesis [46], [48], [58]. Inhibition of translation in cells treated with MG132 was also dependent on the phosphorylation of eIF2α [47]. VSV replication, as well as synthesis of VSV proteins, and most of cellular proteins are sensitive to eIF2α phosphorylation [49], which blocks recycling of this essential component of cap-dependent initiation of translation. VSV infection does not induce eIF2α phosphorylation, at least until the late stage of infection [49].

A plausible explanation of suppression mechanism of VSV replication by proteasome inhibitors involves generation of stress in cells with decreased proteasome activity [47]. In our experiments, HeLa cells treated with MG132 and Bortezomib responded with accumulation of a chaperone protein hsp70, phosphorylation of eIF2α, and suppression of general translation. In agreement with the role of GCN2 in MG132 induced stress [47], the effect of proteasome inhibitors on VSV replication depended on GCN2 activity in the process of stress-related inhibition of translation. GCN2 activity protected fibroblasts from VSV infection [59], and detrimental effect of proteasome inhibitors on VSV replication was stronger in wt GCN2+/+ MEF cells than in GCN2−/− MEF, where MG132-specific attenuation of translation and eIF2α phosphorylation were not efficient. This is the direct indication that stress is the main reason of VSV replication inhibition. The stress may be more profound in MG 132 treated cells infected with VSV. These cells have an abundance of newly synthesized proteins that must be folded with the assistance of chaperones. Proteasome inhibition increased the amount of improperly disposed unfolded cellular and viral proteins. Viral infection may also prevent the additional synthesis of cellular chaperones amplifying stress-related inhibition of translation. This eFI2α-phosphorylation dependent inhibition of translation affects translation of cellular mRNAs as well as VSV mRNAs. Inefficient translation of VSV mRNA decreases the amount of newly synthesized RNA polymerase L, which further affects the synthesis of viral mRNA and proteins. As a result, viral replication decreased up to 100-fold, which protects the cells from some of the toxic effects of VSV infection. Although we did not study the kinetics of VSV replication in MG132 treated cells, even after overnight infection, markers of viral infection were significantly suppressed in cells with decreased proteasome activity. Thus proteasome inhibition may represent a novel therapeutic approach against some viral infections, such as VSV.

In contrast with cellular and VSV protein synthesis, poliovirus protein synthesis was only delayed by proteasome inhibition. Similar delays in replication were reported for Sindbis virus by brefeldin A generated stress [60], and for poliovirus in the cells treated with an inhibitor of the RNA helicase eIF4A [61]. Although the mechanism of the delay is under investigation, the low sensitivity of poliovirus infection to the stress produced by proteasome inhibitor can be due to its IRES-dependent translation. Stress related phosphorylation of eIF2α decreases the general level of translation, but the efficiency of translation of several cellular mRNAs increases during the stress [62]–[64]. Cellular chaperones hsp70 and GRP78, and SNAT2 neutral amino acid transporter are among the genes whose expression increases during such stress [35], [62], [62]–[67]. IRES-dependent initiation of translation is a mechanism to support translation during stress [68], [69]. IRES-dependent translation is less reliant on standard translation initiation factors. Translation from IRES of dicistroviruses does not require any translational initiation factors [70], [71]. Translation from HCV IRES is resistant to decrease in the amount of eIF2α [72], [73]. Poliovirus infection stimulated the phosphorylation of eIF2α [50]. We suggest that IRES-dependent poliovirus translation confers resistance to the inhibition of translation stimulated by stress due to proteasome inhibitor's treatment. Although learning the details of this effect will require additional studies, it may represent a general mechanism of viral stress resistance.

In conclusion, the proteasome inhibition initiated stress-related processes in the cells. These processes included the GCN2-specific phosphorylation of eIF2α, inhibition of general translation, and accumulation of chaperone protein hsp70. Although stress is a general inhibitor of viral replication, its efficacy differs for some viruses. Cap-dependent translation of VSV mRNA is sensitive to the stress, and as a result, the proteasome inhibition had a detrimental effect on VSV replication. In contrast, cap-independent IRES-dependent translation of poliovirus RNA was less sensitive to the stress produced by proteasome inhibitors and by poliovirus replication. As a result, the replication of poliovirus was delayed but not abolished in HeLa cells treated with MG132 and Bortezomib. To further substantiate this explanation, we are studying the effects of similar stresses on other Picornaviruses and Rhabdoviruses.

## Materials and Methods

### Cell culture, DNA transfection, virus infection, and titration

HeLa, wt GCN2+/+ MEF, and GCN2−/− MEF were cultured in Dulbecco modified Eagle's medium (Invitrogen/Gibco BRL) supplemented with 10% fetal calf serum. HeLa cells were infected with poliovirus type 1 Mahoney strain at an input multiplicity of infection (MOI) of 5 plaque-forming units (PFU)/cell for 1h–6 h [74]. HeLa and MEF cells were infected with VSV strain New Jersey with 5 PFU/cell for 1–5 h, or with 1 PFU/cell overnight. For poliovirus titration, 10-fold serial dilutions (1∶10 to 1∶10**^9^**) of the culture medium were added in duplicate to HeLa cells cultured in 48-well plates. Poliovirus titer was determined by the cytopathic effects visible after 3 days. VSV titer was determined in duplicate by plaque assay of 10 fold serial dilutions (1∶10**^4^** to 1∶10**^7^**) of culture medium. MG132 and proteasome inhibitor 1 were obtained from Calbiochem. Bortezomib was provided by Roswell Park Hospital.

### Western immunoblotting

Total protein extracts from HeLa and BHK cells were prepared in RIPA buffer (150 mM NaCl, 1% SDS, 10 mM Tris (pH 8.0), 1% sodium deoxycholate, 1% NP-40) containing a protease inhibitor cocktail (Sigma). Protein extracts were separated by electrophoresis in 4–20% gradient polyacrylamide gels with SDS (Invitrogen/Novex) and then transferred to nylon PVDF membranes (Amersham). The following antibodies were used: anti-VSV P- and N-protein antibodies obtained by immunization of rabbits, anti-protein 3A mouse monoclonal antibodies were the gift from Dr. K. Kirkegaard, anti-protein 3C rabbit antibodies were a gift from Dr. B. L. Semler, anti-poliovirus capsid proteins antibodies obtained by immunization of rabbits with purified poliovirus, anti-p220 eIF4G mouse antibodies were a gift from Dr. T. Pestova, anti-p65-RelA C-terminus rabbit antibodies (Santa Cruz Biotechnology), anti-IκBα rabbit antibodies (Santa Cruz Biotechnology), anti actin rabbit antibodies (Santa Cruz Biotechnology), anti-GCN2 rabbit antibodies (Santa Cruz Biotechnology), and anti-hsp70 rabbit antibodies (Assay Designs/StressGen). Phosphorylation of eIF2α was studied with anti-eIF2α and eIF2α-phospate specific antibodies (Cell Signaling Technology). Immune complexes were visualized by enhanced chemiluminescence (PerkinElmer Life Sciences). The control of protein loading in the gel was done with rabbit anti-Hsp90 antibodies (Abcam, Inc), anti-tubulin rabbit antibodies (Santa Cruz Biotechnology), and anti-keratin 18 rabbit antibodies (a gift from Dr. R. Oshima). HRP-conjugated secondary anti-rabbit and anti-mouse antibodies were purchased from Santa Cruz Biotechnology. Band intensities were quantified using NIH ImageJ software to calculate percentage of protein accumulation (acc), cleavage (cl), phosphorylation (Phosp), or inhibition of protein synthesis (inh).

### Northern blotting

Total RNA from poliovirus or VSV infected HeLa cells were analyzed by Northern blot hybridization with probes specific to poliovirus RNA (3C-coding PCR fragment), VSV P-protein cDNA, and GAPDH gene. A PCR fragment was generated from poliovirus genomic cDNA with the primers specific for poliovirus 3C coding sequence (3Cs 5′ GGG CCT GGG TTT GAC TAT 3′; 3Ca 5′ TTG GCT CTG AGT GAA GTA TGA 3′). The VSV P-protein cDNA probe was generated by PCR from a plasmid containing genomic VSV cDNA with the primers corresponding to 5′ and 3′ ends of P-protein cDNA (P-VSVs 5′ GAC ACA GAA TCT GAA CCA GAA ATT GAA 3′, P-VSVa 5′ TTA TGA GAC ATT CGT CCG TTA CCT CCG 3′). Quantitation of the hybridization signals was done by NIH ImageJ software.

### In vivo S^35^-protein labeling and immunoprecipitation

HeLa cells were infected with VSV or poliovirus for the indicated times and treated with MG132. Regular medium was changed to a methionine/cysteine free medium supplemented with S**^35^** methionine and S^35^ cysteine (50 µCi/ml) (New England Nuclear), and the cells were incubated for 30 min. Cytoplasmic protein extracts from HeLa cells were purified according to the Dignam protocol [34]. Viral and cellular proteins were precipitated from cytoplasmic extracts with corresponding antibodies overnight at 4°C. Antigen/antibody complexes were purified on protein A Sepharose (Sigma) during 1 h incubation. Eluted proteins were analyzed by electrophoresis and autoradiography.

### Poliovirus adsorption and internalization

Poliovirus was labeled by S**^35^**-methionine/cystein during replication. HeLa cells were incubated with labeled poliovirus (MOI = 100) at 4°C for one hour. Control cells were washed 3 times with cold PBS and protein extracts were collected with RIPA. To estimate virus internalization, after incubation with labeled virus at 4°C, medium was changed and cells were transferred to 37°C for additional one hour. Cells were washed 3 times with PBS and protein extracts were collected with RIPA. Presence of S**^35^**-labeled poliovirus capsid proteins were analyzed by electrophoresis and autoradiography. Band intensities were quantified using NIH ImageJ software to calculate percentage of protein adsorption (ad) and internalization (in).
